# The T2T-CHM13 reference assembly uncovers essential WASH1 and GPRIN2 paralogues

**DOI:** 10.1093/bioadv/vbae029

**Published:** 2024-02-28

**Authors:** Daniel Cerdán-Vélez, Michael Liam Tress

**Affiliations:** Bioinformatics Unit, Spanish National Cancer Research Centre (CNIO), Madrid 28029, Spain; Bioinformatics Unit, Spanish National Cancer Research Centre (CNIO), Madrid 28029, Spain

## Abstract

**Summary:**

The recently published T2T-CHM13 reference assembly completed the annotation of the final 8% of the human genome. It introduced 1956 genes, close to 100 of which are predicted to be coding because they have a protein coding parent gene. Here, we confirm the coding status and functional relevance of two of these genes, paralogues of *WASHC1* and *GPRIN2*. We find that *LOC124908094*, one of four novel subtelomeric WASH1 genes uncovered in the new assembly, produces the WASH1 protein that forms part of the vital actin-regulatory WASH complex. Its coding status is supported by abundant proteomics, conservation, and cDNA evidence. It was previously assumed that gene *WASHC1* produced the functional WASH1 protein, but new evidence shows that *WASHC1* is a human-derived duplication and likely to be one of 12 WASH1 pseudogenes in the human gene set. We also find that the T2T-CHM13 assembly has added a functionally important copy of *GPRIN2* to the human gene set. We demonstrate that uniquely mapping peptides from proteomics databases support the novel *LOC124900631* rather than the GRCh38 assembly *GPRIN2* gene. These new additions to the set of human coding genes underlines the importance of the new T2T-CHM13 assembly.

**Availability and implementation:**

None.

## 1 Introduction

The publication of the T2T-CHM13 genome assembly by the Telomere-to-Telomere consortium (Nurk *et al.* 2022) addressed the 8% of the human genome that was missing in the GRCh38 reference. The new gapless assembly added 1956 gene predictions, 140 of which were similar to known coding genes. It is not clear how many of these 140 predicted coding genes can code for proteins. Most are recent duplications, and the consortium did not assess coding potential. Many recent gene duplications are likely to be either pseudogenes or in the process of pseudogenization (Lynch and Conery 2000, Wagner 2001).

The T2T-CHM13 genome assembly uncovered four novel *WASHC1* paralogues in newly annotated subtelomeric regions on chromosomes 3, 11, and 20. Nine *WASHC1* paralogues were already annotated in the GRCh38 human reference set: two pseudogenes on the p arm of chromosome 1, paralogues on chromosomes 2, 9, 12, 15, 16, and 19, and the pseudoautosomal regions of X and Y. Eight are found in subtelomeric regions, while the ninth, *WASH2P*, is present in the ancestral telomere-telomere fusion site on chromosome 2 (IJdo *et al.* 1991).

*WASHC1* is thought to be the gene that produces the WASH1 protein, one of the components of the WASH (Wiskott-Aldrich syndrome protein and SCAR Homologue) complex. The WASH complex is one of several that activate the Arp2/3 complex at different subcellular locations (Alekhina et al. 2017). The activated Arp2/3 complex has a crucial role in the generation of branched actin filaments and is important in a range of processes including endocytosis, intracellular trafficking and membrane remodelling. The WASH complex plays a key role in endosomal sorting by recruiting the Arp2/3 complex to induce actin polymerization (Derivery *et al.* 2009, Gomez and Billadeau 2009). The WASH complex has been shown to be essential in both *Drosophila* (Linardopoulou *et al.* 2007) and mice (Xia *et al.* 2014), and defects in its components lead to inherited developmental and neurological disorders (Valdmanis *et al.* 2007, Courtland *et al.* 2021).

The WASH complex is made up of proteins from five genes, *WASHC1* (previously *WASH1*), *WASHC2A* (*FAM21A*), *WASHC3* (*CCDC53*), *WASHC4* (*SWIP*), and *WASHC5* (*KIAA0196*). The exact composition of the WASH complex in great apes is complicated by the presence of two highly similar FAM21 genes*, WASHC2A* and *WASHC2C*. The two FAM21 genes were generated by a duplication and translocation on chromosome 10 between q11.22 and q11.23, apparently in the ancestor of humans and chimpanzees. In humans, both *WASHC2A* and *WASHC2C* have clear peptide support.

In addition, humans, chimpanzees and gorillas have multiple WASH1 genes. In great apes, WASH1 genes are found in rearrangement-prone subtelomeric regions near the ends of chromosomes (Linardopoulou *et al.* 2007), and gene duplications from interchromosomal duplication are common in subtelomeric regions.

Most of the nine WASH1 genes currently annotated in the GRCh38 assembly are either not full length or have premature stop codons or frameshifts that will likely render them inactive (Linardopoulou *et al.* 2007). Just one gene, *WASHC1* on chromosome 9, is full length and intact. The other eight genes have names that mark them out as pseudogenes, from *WASH2P* (on chromosome 2) to *WASH9P* (chromosome 1).

There is disagreement between the three main reference databases as to which WASH1 genes code for proteins. RefSeq (Sayers *et al.* 2023a) annotates *WASHC1* alone as coding, but Ensembl/GENCODE (Frankish *et al.* 2023, Martin *et al.* 2023) predicts that both *WASHC1* and *WASH6P* (chromosomes X/Y) are coding. Finally, UniProtKB (The UniProtKB Consortium 2023) lists isoforms for *WASHC1*, *WASH2P*, *WASH3P* (chromosome 15), *WASH4P* (chromosome 16), and *WASH6P*. *WASH4P* and *WASH6P* lost their 5ʹ ends when duplicated, so do not produce the whole WASH1 protein, *WASH2P* contains two premature stop codons, and *WASH3P* has a 4-base frame-shifting deletion and a premature stop codon.

Part of the reason for the disagreements between reference sets is that much of the peptide evidence from proteomics experiments maps to genes other than *WASHC1*, suggesting that multiple WASH1 paralogues exist. In addition, the WASH1 cDNA (BC048328.1) produced by the Mammalian Gene Collection Program (Strausberg *et al.* 2002) is quite distinct from the cDNA expected from *WASHC1*. It is notable that experiments investigating the function of the WASH complex and WASH1 have tended not to use human *WASHC1*. For example, the initial experiments that discovered the function of the WASH complex used murine WASH1 (Derivery *et al.* 2009), or the BC048328.1 cDNA product (Linardopoulou *et al.* 2007, Gomez and Billadeau 2009).

The ancestral WASH1 gene in apes is predicted to be located on the p arm of chromosome 12; probes used in distinct primate species detected WASH1 genes here in macaque, orangutan, gorilla, and chimpanzee, as well as human (Linardopoulou *et al.* 2007). Multiple WASH1 paralogues have been detected in subtelomeric regions in the gorilla, chimpanzee and human genomes (Linardopoulou *et al.* 2007). Along with the ancestral gene on chromosome 12 (*WASH8P* in the GRCh38 human genome assembly), a second WASH1 gene on the p arm of chromosome 20 was also detected in these three species (Linardopoulou *et al.* 2007). This gene is not present in the GRCh38 assembly.

The T2T-CHM13 genome assembly also uncovered a novel *GPRIN2* gene, *LOC124900631.* By way of contrast to *WASHC1*, less is known about gene *GPRIN2* (G protein-regulated inducer of neurite outgrowth 2). *GPRIN2* was found to interact with G-proteins from *GNAO1* (Chen *et al.* 1999) and induced morphological changes to neurites. *GPRIN2* has been highlighted as an illustrative example of copy number variation (CNV) in several recent publications (Handsaker *et al.* 2015, Vollger *et al.* 2022, Liao *et al.* 2023). In these papers *GPRIN2* was found to be present in two copies in most individuals.

Here, we show that one of the two novel full length WASH1 genes annotated on chromosome 20 as part of the new T2T-CHM13 reference assembly is likely to produce the WASH complex protein, and that the newly annotated *GPRIN2* gene is likely to be the protein involved in the regulation of neurite growth.

## 2 Methods

### 2.1 Sequence analysis

Predicted protein sequences for *WASHC1*, *WASH2P*, *WASH3P*, *WASH4P*, and *WASH6P* were obtained from UniProtKB. The predicted protein sequences of XP_047302880.1 and XP_047302896.1 from WASH1 genes *LOC124908094* and *LOC124908102* were downloaded from RefSeq.

For the *GPRIN2* analyses, the sequence of XP_047301623.1 from *LOC124900631* was downloaded from RefSeq, and the principal isoforms from *GPRIN2*, *SYT15*, *SYT15B*, *NPY4R*, and *NPY4R2* were downloaded from APPRIS (Rodriguez *et al.* 2022).

We downloaded the protein sequence generated from clones BC048328.1 (for *WASHC1*) and BAA25440.2 (*GPRIN2*) from GenBank (Sayers *et al.* 2023b).

The alignment of vertebrate *WASHC1* proteins was carried out using UniProtKB *WASHC1* protein sequences from 27 vertebrate species plus the human WASH1 protein sequences from in UniProtKB and XP_047302880.1 from *LOC124908094*. The only requirement for inclusion in the alignment was that the all sequences had to have the ancestral N- and C-terminal sequences and no insertions or deletions of >7 amino acid residues relative to the human *WASHC1* sequences. We included sequences from multiple eutherian, therian, bird, reptile, amphibian, and fish species as well as *WASHC1* sequences from ghost shark and European lancelet.

### 2.2 Peptide mapping

We downloaded the peptides detected for all WASH1 and GPRIN2 proteins in the current version (2023–01) of the PeptideAtlas database (Kusebauch *et al.* 2014). These genes that produce these proteins include *WASHC1* and *GPRIN2*, but also *WASH2P*, *WASH3P*, *WASH4P*, and *WASH6P* because UniProtKB predicts proteins for these genes. PeptideAtlas also maps peptides to protein sequences from the T2T-CHM13 assembly with RefSeq coding sequences (*LOC124908094*, *LOC124908102*, and *LOC124900631*) and includes peptides that are single amino acid variations of the peptides that map to these proteins.

PeptideAtlas carries out a reanalysis of spectra from multiple large- and small-scale proteomics experiments using state-of-the-art search engines before post-processing with the in-house trans-proteomic pipeline (Deutsch *et al.* 2023). Each peptide may be detected multiple times over the distinct experiments. PeptideAtlas records the number of distinct “observations” in which a peptide is detected. We used these observations as a rough measure of the expression level of each peptide.

To be sure that we were working with higher quality peptides, we required that all peptides had at least two observations (we removed all singletons), and that each peptide was fully tryptic and had no more than two missed lysine or arginine cleavages.

For the WASH1 analysis, we remapped the peptides recorded in PeptideAtlas to the five WASH1 proteins annotated in UniProtKB, and to the predicted sequence of XP_047302880.1 from gene *LOC124908094*. This gene is located on chromosome 20 at the terminus of the p arm of chromosome 20. The predicted protein XP_047302880.1 is referred to throughout the paper as WASH1-20p13.

For the GPRIN2 analysis, we mapped the peptides recorded in PeptideAtlas to the *GPRIN2* principal isoform ENST00000374314.4 and to XP_047301623.1.

### 2.3 Phylogenetic analysis

For the WASH1 phylogenetic tree we included the full length WASH1 genes annotated in human, chimpanzee, pygmy chimpanzee (bonobo), gorilla, orangutan, and gibbon. These included six of the nine WASH1 genes annotated in the GRCh38 assembly of the human genome (*WASH4P*, *WASH5P* and *WASH6P* were left out of the analysis because they are missing exons) and the four WASH1 genes added in the T2T-CHM13 assembly. Both chimpanzee (*LOC741303*, *LOC456647*) and bonobo (*LOC117978042*, *LOC117975334*) are annotated with two WASH1 genes; in both species the transcripts are full length and located on chromosomes 12 and 20. Gorilla is annotated with multiple WASH1 genes, but only one is full length and without stop codons (*LOC109025132*). Orangutan (*WASHC1*) and gibbon (*WASHC1*) are annotated with a single WASH1 gene each.

The DNA sequences of novel WASH1 transcripts XR_007073267.1, XM_047445807.1, XM_047446924.1, and XM_047446940.1 from *LOC124906207*, *LOC124906931*, *LOC124908094* and *LOC124908102* were downloaded from RefSeq along with transcripts with equivalent exon combinations for *WASHC1*, *WASH2P*, *WASH3P*, *WASH7P*, *WASH8P* and *WASH9P.* We added chimpanzee (XM_024353717.2, XM_024347602.2), bonobo (XM_055104758.1, XM_034935049.2), gorilla (XM_031011818.2), orangutan (XM_009237661.3), and gibbon (XM_032159216.2) *WASHC1* transcripts. We created the phylogenetic tree of the WASH1 genes from the aligned coding exons and introns of the 17 human and primate WASH1 genes.

The phylogenetic tree was generated with Phyml 3.0 with 100 bootstrap replicates (Guindon *et al.* 2010) using the TN93+I substitution model according to SMS (Lefort *et al.* 2017) and running 1000 non-parametric bootstrap replicas. The gibbon *WASHC1* sequence was forced to root the tree in the analysis.

## 3 Results

### 3.1 Novel WASH1 paralogues are found in subtelomeric regions

**Figure 1.**
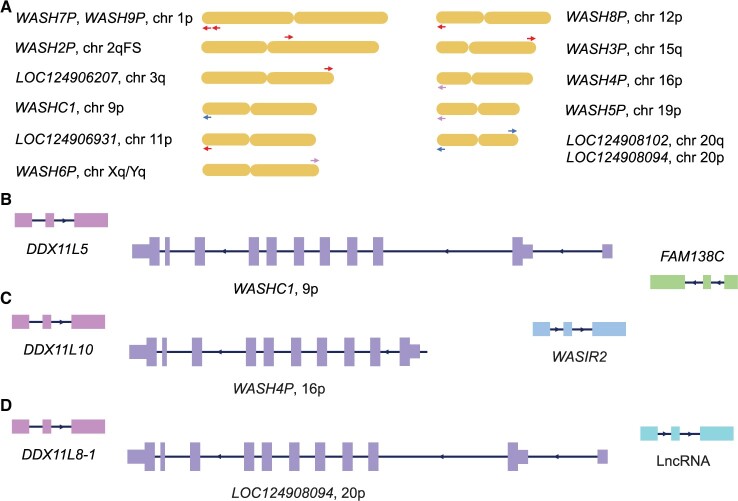
Annotated human WASH1 genes and WASH1 gene neighbourhoods. (A) The 13 WASH1 genes in the human genome and their approximate position in the chromosome. Chromosomes are represented by a single chromatid and are not to scale. The approximate position and the direction of the labelled WASH1 gene is indicated with an arrow. Genes represented with red arrows have premature stops and/or frameshift mutations, genes represented by pink arrows cannot produce the full WASH1 protein, genes represented by blue arrows are full length and without high impact mutations. Genes are positioned on the p arm (p), the q arm (q), or the ancestral fusion site (FS). The p arm of chromosome 1 has two WASH1 genes on the same strand. (B) The gene neighbours of *WASHC1* on chromosome 9. Exons (blocks) and introns not to scale. Coding exons are wider than non-coding exons. Direction of the strand is shown by the arrows in the intronic regions. The DDX11-like pseudogene is on the left. The FAM138 gene on the right. This block spans the chromosome 9 region from approximately base 12 000 to base 37 500. Most human WASH1 genes have this gene neighbourhood. (C) The gene neighbours of *WASH4P* on chromosome 16. *WASH4P* is missing its 5ʹ end and is predicted to translate from an upstream start site in an L2 transposon region. The DDX11-like pseudogene is on the left. The WASIR gene on the right. (D) The gene neighbours of *LOC124908094* on chromosome 20. Thje DDX11-like pseudogene is on the left, lncRNA exons on the right.

All four genes newly annotated in the T2T-CHM13 assembly are located at the subtelomeric regions at the terminal end of chromosomes (Fig. 1A). Subtelomeric regions are prone to rearrangement and interchromosomal duplications affect gene content (Linardopoulou *et al.* 2005). Two of the novel WASH1 genes would produce full length proteins, both are on chromosome 20.

In total just three WASH1 genes could produce full length proteins. The remainder either have frameshifts or premature stop codons that would truncate the protein (*WASH2P*, *WASH3P*, *WASH7P*, *WASH8P*, *WASH9P*, *LOC124906207, LOC124906931*) or would not produce a complete WASH1 protein (*WASH4P*, *WASH5P, WASH6P*). *WASH4P* and *WASH6P* have lost the 5ʹ coding exon (this exon codes for 50 amino acids). *WASH5P* is missing most of its coding exons in the GRCh38 assembly. This region has been completed in the T2T-CHM13 assembly, though exons are still unannotated. The WASH1 genes analysed in this work are shown in Table 1 and in Supplementary Table S1.

**Table 1.** Annotated and novel WASH1 and GPRIN2 genes and pseudogenes.^a^

Gene symbol	Chr	Assembly	Alias	Predicted
*WASHC1*	9	GRCh38		Pseudogene
*WASH2P*	2	GRCh38		Pseudogene
*WASH3P*	15	GRCh38		Pseudogene
*WASH4P*	16	GRCh38		Pseudogene
*WASH5P*	19	GRCh38		Pseudogene
*WASH6P*	X	GRCh38		Pseudogene
*WASH7P*	1	GRCh38		Pseudogene
*WASH8P*	12	GRCh38		Pseudogene
*WASH9P*	1	GRCh38		Pseudogene
*LOC124908094*	20	CHM13	WASH1-20p13	Coding
*LOC124908102*	20	CHM13	WASH1-20q33	Pseudogene
*LOC124906207*	3	CHM13		Pseudogene
*LOC124906931*	11	CHM13		Pseudogene
*GPRIN2*	10	GRCh38	.	Coding
*LOC124900631*	10	CHM13	*GPRIN2L*	Coding

a A list of the 10 GRCh38 and 5 T2T-CHM13 assembly genes mentioned in this paper, along with any aliases used and whether we predict these genes are pseudogenes or coding genes. There is an expanded version of this paper in the supplementary material.

### 3.2 Annotated WASH1 genes have a similar gene neighbourhood

The 13 WASH1 paralogues are a result of inter-chromosomal duplications in the subtelomeric regions, duplications that involve various genes in a single block. As a result, most WASH1 genes have similar gene neighbours. These duplicated blocks seem to be of different sizes, but even those genes where only part of the WASH1 gene was duplicated (*WASH4P*, *WASH6P*) have retained their gene neighbours on one side at least.

Most WASH1 genes are preceded upstream on the same strand by a pseudogene from the FAM138 family (Fig. 1B). FAM138 genes are non-coding in the human gene set but annotated as coding in some primate species. Just downstream of the WASH1 genes, and on the opposite strand, is a DDX11-like gene, a probable pseudogene made up of a short fragment of a dead-box helicase gene.

Eight of the 13 WASH1 genes have the *WASH1C* gene neighbourhood. The exceptions include *WASH4P* and *WASH6P*, which were duplicated without their 5ʹ exons and without the FAM138 family gene beyond the 5ʹ exons. Instead, *WASH4P* and *WASH6P* have upstream WASIR genes (Fig. 1C). *WASH5P* is missing the DDX11-like gene in GRCh38 because of a gap in the assembly, and *WASH9P* is missing the FAM138 gene neighbour for the same reason. In the T2T-CHM13 assembly, *WASH5P* does have a neighbouring DDX11-like gene, but *WASH5P* is not annotated with all its exons. Neither *WASH9P* nor its neighbours are yet annotated in the T2T-CHM13 assembly. Finally, *LOC124908094*, on the p arm of chromosome 20, is missing the FAM138 family gene (Fig. 1D).

At present, the reference sets of bonobo and chimpanzee are annotated with two copies of WASH1 genes each. In chimpanzee and bonobo, WASH1 genes are annotated on both chromosome 12 and chromosome 20. In both species, the chromosome 12 WASH1 gene neighbourhood is bounded by a DDX11-like pseudogene and a FAM138 family gene, just like the equivalent human WASH1 gene on chromosome 12 (Fig. 1B). The chimpanzee WASH1 paralogue (*LOC741303*) on chromosome 20 is adjacent to just the DDX11-like pseudogene. As with *LOC124908094* on human chromosome 20, there is no upstream FAM138 family gene (Fig. 1C). However, both *LOC741303* and *LOC117978042* (the bonobo WASH1 gene on chromosome 20) do have a *DEFB125* coding gene a little further upstream, just like the human chromosome 20 paralogue, *LOC124908094.*

### 3.3 The WASH1 paralogue on chromosome 12 is the ancestral *WASHC1* gene

Curiously, the association between the *WASHC1* gene and DDX11-like helicases has ancient roots. Zebrafish has a single *wash1* gene, and the full length *ddx11* gene is downstream and on the opposite strand. When the ancestral *WASHC1* gene ended up in the subtelomeric regions, it seems it only retained part of the *DDX11* gene.

The ancestral *WASHC1* gene in human is clearly the chromosome 12 WASH1 gene (*WASH8P*). *WASH8P* has an antisense *IQSEC3* coding gene just upstream, beyond which are *SLC6A12*, *SLC6A13*, and *KDM5A* coding genes on the same strand as *WASHC1*. This is also true of the chromosome 12 *WASHC1* genes in chimpanzee, bonobo, gorilla and orangutan. In fact, the *FKBP4*-*DDX11*-*WASHC1*-*IQSEC3*-*SLC6A12*/*SLC6A13*-*KDM5A* gene block is contiguous across tetrapods, while *IQSEC3* is a neighbour of *WASHC1* even in elephant shark.

It appears that a block of genes that included *DDX11* and *WASH1C* was duplicated at some point in the ancestor of the Euarchontoglires clade and this allowed species to rearrange the order of these genes. In primates, a block of 16 coding genes (including *WASHC1*, *IQSEC3*, *SLC6A12*, *SLC6A13*, and *KDM5A*) at the end of the chromosome has been reversed with respect to its ancestral direction. The block ended in middle of *DDX11*, leaving *WASHC1* and the *DDX11* fragment at the border of the reversed region. *WASHC1* and the *DDX11* fragment are part of or close to the subtelomeric region in almost all old-world monkeys. In humans the *DDX11* coding gene is still in the p arm of chromosome 12 but is now close to the centromere.

### 3.4 Cross-species conservation supports the importance of *LOC124908094* protein WASH1-20p13

UniProtKB annotates five WASH1 proteins. Two of these predicted proteins (from *WASH2P* and *WASH3P*) would be truncated because they have premature stop codons and frameshifts relative to the ancestral *WASHC1* gene. Two more (from *WASH4P* and *WASH6P*) are predicted to initiate their translation from different frames of the same non-conserved L2 transposon region (Fig. 1C) and would be very different from ancestral WASH1 protein in the N-terminal region. In addition, alignments with WASH1 proteins from vertebrate species show that all five UniProtKB WASH1 proteins have multiple single amino acid differences in residues that are conserved across mammals and tetrapods (Supplementary Fig. S1).

**Figure 2.**
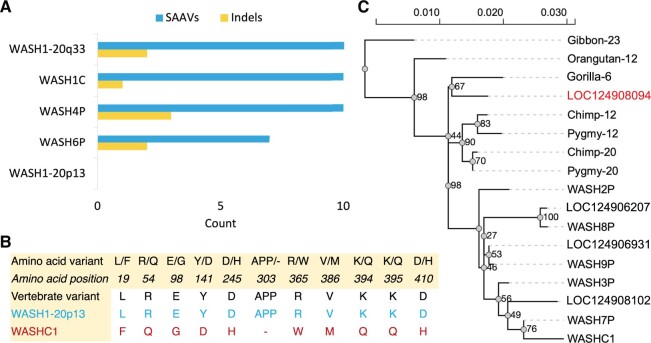
WASH1 variants and phylogenetic analysis. (A) The number of non-conserved amino acids (single amino acid variations, SAAVs) and deleted regions in the five full length WASH1 protein isoforms that differ from amino acids that are conserved across primates, mammals and tetrapods. WASH1-20q33 is the predicted protein on the q arm of chromosome 20. WASH1-20p13 is left blank because it only differs in non-conserved regions. (B) Amino acid differences between the *WASHC1* protein and WASH1-20p13 in regions conserved across vertebrates. The *WASHC1* protein is different in all conserved positions. (C) Phylogenetic tree of great ape and human genes. Genes newly annotated in T2T-CHM13 assembly are labelled with their RefSeq gene names, the likely WASH1 gene, *LOC124908094*, is highlighted. Great ape WASH1 genes are labelled with the chromosome number in which they are annotated.

However, one WASH1 protein has largely maintained the conserved ancestral WASH1 sequence, the predicted full length WASH1 protein from *LOC124908094* on the p arm of chromosome 20, XP_047302880.1. This protein is referred to as “WASH1-20p13” in the remainder of the text. Not only is WASH1-20p13 the only WASH1 isoform that maintains amino acids conserved across vertebrates, but it is also the only isoform without any deletions (Fig. 2A). All WASH1 proteins other than WASH1-20p13 have a three-residue deletion with respect to vertebrate WASH1 sequences.

Most of the amino acid substitutions in annotated UniProtKB WASH1 proteins are not conservative. For example, none of the 10 single amino acid differences between the *WASHC1* principal isoform and WASH1-20p13 that fall in conserved regions are conservative (Taylor 1986). The ten differences between the *WASHC1* principal isoform and WASH1-20p13 can be seen in Fig. 2B.

Several amino acid substitutions in *WASHC1* might even be considered radical (Taylor 1986). One example is the tyrosine to aspartate swap at residue 141. Tyrosine residue Y141 is phosphorylated by the Src kinase LCK and mutating this residue to phenylalanine has been shown to interfere with WASH-mediated NK cell cytotoxicity (Huang *et al.* 2016). Since aspartate is a phosphomimetic, the effect of a Y141D mutation may well be permanent phosphorylation, leading to sustained intensification of NK cell cytotoxicity.

We carried out a phylogenetic analysis with ten of the WASH1 paralogues and annotated WASH1 genes in great apes. The analysis confirms that *WASHC1*, *WASH2P*, *WASH3P*, *WASH7P*, *WASH8P*, *WASH9P*, *LOC124906207*, *LOC124906931*, and *LOC124908102* all derived from a common ancestor because they form a separate outgroup (Fig. 2C). *WASH4P* and *WASH6P* were not included in the analysis because of the large 5ʹ region differences, but their common variants and indels suggest that they almost certainly derived from the same common ancestor. *WASH5P* has too little sequence to include in the analysis. *LOC124908094* from chromosome 20 grouped with the primate WASH1 genes.

### 3.5 Peptide evidence does not support the currently annotated *WASHC1* coding gene

To determine which WASH1 genes had translation evidence, we mapped all peptides recorded in PeptideAtlas to the five WASH1 isoforms annotated in UniProtKB, and to the full length WASH1 protein from *LOC124908094*, WASH1-20p13.

**Figure 3.**
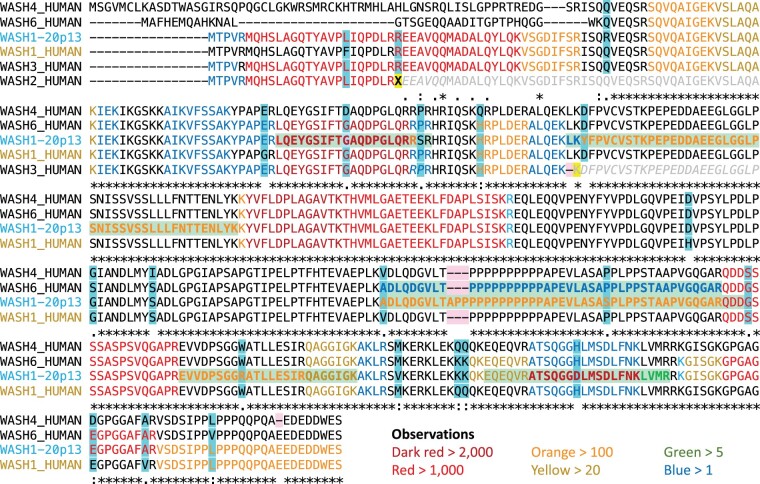
Mapping peptides to WASH1-20p13 and WASH1 sequences in UniProtKB. Peptides are mapped to alignments between the five UniProtKB annotated WASH1 sequences and the newly annotated WASH1-20p13 protein. Residues that peptides map to are colour coded by the number of observations detected for that protein. Dark red, red, and orange fonts indicate the most observed peptides. Residues with a yellow background indicate the position of stop codons and frameshifts in *WASH2P* and *WASH3P*. Grey sequence indicates the parts of the predicted sequence of *WASH2P* and *WASH3P* that cannot be translated because of these stop codons and frameshifts. Peptides with a light green background and text in bold show those peptides that map uniquely to one of the six sequences. Amino acids columns with a blue background indicate the position of single amino acid differences between the predicted proteins. Deletions relative to ancestral sequences are marked with a pink background.

In total, 52 peptides with a total of 23 813 observations in PeptideAtlas map to at least one of the six WASH1 isoforms. The peptides, coloured by the number of observations, are mapped onto the predicted sequences of the six proteins in Fig. 3. None of the WASH1 isoforms captures all 52 of the PeptideAtlas peptides, at first glance suggesting that more than one WASH1 gene might be coding.

*WASHC1*, the only gene annotated as coding in all three main human reference databases, captures surprisingly few peptides. Just half of the 52 PeptideAtlas peptides map to the *WASHC1* isoform. Ensembl/GENCODE annotates *WASH6P* and *WASHC1* as coding, but *WASH6P* and *WASHC1* between them capture only 34 PeptideAtlas peptides. This leaves more than a third of PeptideAtlas WASH1 peptides unaccounted for.

Extending the mapping to predicted UniProtKB proteins from *WASH2P*, *WASH3P*, and *WASH4P* would cover most of the 52 WASH1 peptides in PeptideAtlas, if the predicted sequences of *WASH2P* and *WASH3P* in UniProtKB were correct. Unfortunately, *WASH3P* has a frameshift that would eliminate the final two thirds of the protein, and *WASH2P* has two premature stop codons, the first of which would leave a rump of just 25 amino acid residues. These two genes cannot produce the predicted proteins.

Of the two full length WASH1 genes identified in the T2T-CHM13 assembly on chromosome 20, the protein from the WASH1 paralogue on the q arm has little support; just 18 of the 52 PeptideAtlas peptides mapped to this protein. By way of contrast, the *LOC124908094* isoform, WASH1-20p13, captures 47 of the 52 PeptideAtlas peptides. In fact, 17 peptides map to WASH1-20p13 and to no other paralogue. Two of these peptides are among the most highly observed, ATSQGGDLMSDLFNK, with >2000 observations, and MQHSLAGQTYAVPLIQPDLR, with 1300 observations. The 17 peptides that map uniquely to WASH1-20p13 strongly suggest that *LOC124908094* produces the WASH complex protein. In PeptideAtlas most observations are from a range of cell lines or cancer cells. However, peptides unique to WASH1-20p13 were detected in all major tissue types, as would be expected from a protein that is ubiquitously expressed (Uhlén *et al.* 2015).

### 3.6 G343S is a common variant in WASH1-20p13

Five of the 52 WASH1 peptides in PeptideAtlas do not map to WASH1-20p13. There are three possible explanations for these unmapped peptide spectrum matches (PSMs). Either WASH1-20p13 has one or more common amino acid variants, two (or more) WASH1 genes are translated, or the PSMs are false positive matches. No single WASH1 isoform captures all five of these peptides. Four of the 5 peptides have 4 or fewer observations, but one peptide, QDDSSSSASPSVQGAPR, was detected 1239 times in experiments across PeptideAtlas. A similar peptide, QDDGSSSASPSVQGAPR, which does map to WASH1-20p13, was observed 1999 times.

It is curious that peptide QDDSSSSASPSVQGAPR has so many observations when none of the other four peptides that do not map to WASH1-20p13 have >4. One possibility is that there is a common variant in WASH1-20p13 that changes glycine residue 343 into a serine. It is noticeable that the only difference between WASH1-20p13 and the protein produced by the BC048328.1 cDNA clone is the same glycine to serine substitution.

Here, the annotations from the Human Pangenome Reference Consortium (Liao *et al.* 2023) were particularly useful. The Human Pangenome Reference Consortium annotation is based on the T2T-CHM13 assembly, so individuals are annotated with the T2T-CHM13 WASH1 genes as well as those only present in the GRCh38 assembly. Individuals are not annotated with all 13 paralogues. We were able to retrieve 33 predicted protein sequences that were most similar to the WASH1-20p13 (those sequences with the ancestral “APP” insertion). Of these, 17 have a glycine at position 343 and 16 have a serine. G343S is quite clearly a common WASH1-20p13 variant. A second variant, S119P, that explains another of the undetected peptides is also present though less common in these 33 sequences. Between them, these two variants explain three of the peptides that do not map to WASH1-20p13. So, all but two of the 52 PeptideAtlas peptides, and 23 808 of the 23 813 peptide observations (99.98%), map to WASH1-20p13 and its common variants.

The remaining two peptides, with a total of five observations, map to *WASH6P*. Only one also maps to the previously predicted WASH1 gene, *WASHC1*. These peptides were detected in cancer cells or cell lines, so there is no evidence that *WASH6P* (if translated) is tissue specific. It seems unlikely that these peptides are from *WASH6P* though, because *WASH6P* has a large novel N-terminal for which we find no supporting peptides at all. Of the two peptides, one (with just two observations and unconvincing peptide spectrum matches) is likely to be a false positive match. The other, which has three observations, is a single amino acid variant of the WASH1-20p13 peptide ATSQGGDLMSDLFNK which has 2024 observations.

### 3.7 *LOC124908094* is likely to be the only WASH1 coding gene

There are 13 WASH1 genes annotated in the T2T-CHM13 assembly. *WASH8P*, the ancestral copy of WASH1 on chromosome 12, is no longer functional because it would generate a transcript with a premature stop codon. This stop codon is present in another five human WASH1 pseudogenes, *WASH3P*, *WASH9P*, *WASH7P, LOC124906931*, the copy of WASH1 on chromosome 11, and *LOC124906207*, the copy of WASH1 on chromosome 3. In addition, *WASH2P* has multiple disabling mutations and *WASH5P*, as annotated, is just a fragment of the whole gene. These eight WASH1 paralogues are clearly pseudogenes.

There are clues that all paralogues apart from *LOC124908094* on chromosome 20 derived from the *WASH8P* pseudogene. FAM138 pseudogenes are found upstream of the chromosome 12 WASH1 genes in both human and chimpanzee, but not upstream of the chromosome 20 WASH1 genes. Instead, chromosome 20 WASH1 genes have an upstream *DEFB125* gene. Nine of the 12 human WASH1 paralogues of *LOC124908094* have upstream FAM138 family pseudogenes. Two of the paralogues without a FAM138-like pseudogene at the 5ʹ end are *WASH4P* and *WASH6P*, but these two genes have also lost their 5ʹ coding exons. The other is *WASH9P*, where the position of the FAM138 gene coincides with a gap in the GRCh38 assembly.

In order to generate proteins for the WASH1 paralogues without peptide support, we would have to ignore frameshifts and stop codons. However, if it were possible to produce proteins from these genes, 11 of the 12 would produce proteins with a 3 amino acid deletion at the start of the proline-rich region and have four single amino acid differences in common, R54Q, Y141D/E, V386M, and D410H. The odd one out, *WASH5P*, would produce a highly truncated protein so was not analysed. None of these five radical changes are present in WASH1 proteins of any other species, not even gorilla or chimpanzee, reinforcing the suggestion that these WASH1 paralogues arose in the human lineage.

Finally, the phylogenetic analysis of the ancestral WASH1 genes confirms that human WASH1 paralogues apart from *LOC124908094* have a common ancestor because they form an outgroup separate from the primate WASH1 genes and *LOC124908094*. While the phylogenetic analysis does not wholly confirm the theory that these 12 WASH1 genes arose in the human lineage, the alternative possibility, that they appeared earlier and have either been lost from other primate species or have not yet been annotated, is highly unlikely.

One curious result from the analysis is that the bonobo (pygmy chimpanzee) and chimpanzee WASH1 genes from chromosomes 12 and 20 form their own outgroup. Alignments between the annotated WASH1 isoforms in chimpanzee and bonobo (Supplementary Fig. S2) reveal few amino acid residue differences. It is possible that chromosome 12 and 20 WASH1 paralogues arose independently in humans and chimpanzees, even though human and chimpanzee WASH1 gene neighbourhoods are similar for both genes. Although this muddies the water regarding WASH1 evolution in subtelomeric regions in primates, it does not affect the evolutionary history of the human WASH1 paralogues.

### 3.8 The T2T assembly uncovers a *GPRIN2* paralogue

The T2T assembly has added >3600 predicted genes and 140 of these genes were found to be most similar to coding genes. Yet similarity to a coding gene is not a guarantee that the duplication itself is coding. These genes have yet to undergo manual curation, and most duplications tend to lose coding ability over time and become pseudogenes (Lynch and Conery 2000, Wagner 2001). No fewer than 57 of the 140 potential coding genes come from just 3 parents—*TAF11L5*, *USP17L11*, and *USP17L27*. We found that at least one of these potential coding genes is coding.

**Figure 4.**
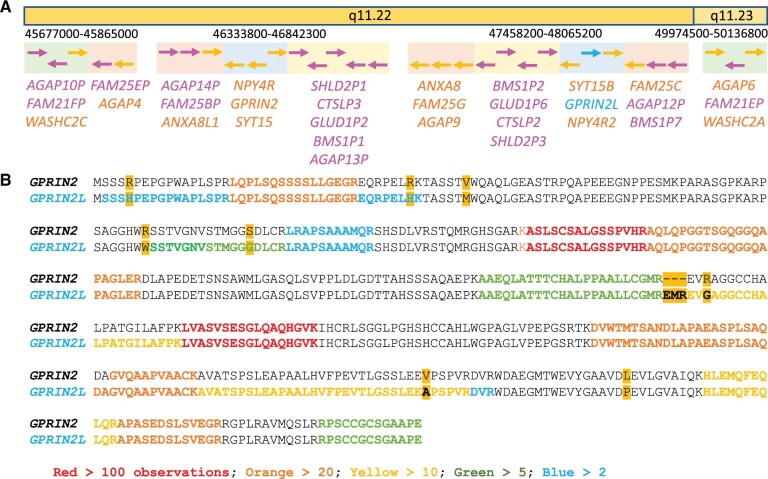
Segmental duplications on chromosome 10 and *GPRIN2* peptides. (A) Duplication and translocation in the q arm of chromosome 10. At least four gene blocks have duplicated in this region since the last common ancestor of humans and chimpanzees. Genes are shown as arrows indicating direction of strand, coding genes are orange arrows, pseudogenes pink. The *GPRIN2L* paralogue uncovered by the T2T-CHM13 assembly is shown in light blue. The four gene blocks that have duplicated are colour coded. The light green background blocks include the WASH2 genes, the light blue background the GPRIN2 paralogues. The AGAP-FAM25 block (orange background) appears to have duplicated three times with multiple genes in this region alone. Genes in the yellow background block are all pseudogenes. Several of the gene blocks are contiguous. There is no gap between the gene blocks when the blocks are contiguous. The approximate coordinates of the contiguous blocks are indicated above the blocks. (B) Alignment of the two human GPRIN2 proteins. The differences between the sequences are highlighted in orange. Peptides detected in PeptideAtlas are mapped onto the sequences. Red peptides have >100 observations, orange >20 observations, yellow >10, green >5, and blue >2. The newly annotated gene, *GPRIN2L* (*LOC124900631*), is supported by six unique peptides with a total of 43 observations.

*LOC124900631* is a duplication of gen*e GPRIN2* (G protein-regulated inducer of neurite outgrowth), a gene annotated on the reverse strand of region q11.22 on chromosome 10 (Deloukas *et al.* 2004). The nearest downstream coding gene is *NPY4R* (Neuropeptide Y receptor type 4), the nearest upstream gene is *SYT15* (Synaptotagmin-15). This block is one of four blocks of genes in the proximal end of the q arm of chromosome 10 to have undergone recent duplication and translocation (Fig. 4A). A region of approximately 0.5MB at the end of q11.22 has been duplicated and inserted back into q11.22, but in the reverse direction. Curiously, the duplicated WASH2 genes are generated from one of the other three duplicated blocks (Fig. 4A). In GRCh38, this segmental duplication region was well characterized (Deloukas *et al.* 2004, Vollger *et al.* 2022) except for a short, gapped region between genes *SYT15B* and *NPY4R2*. The T2T-CHM13 assembly adds one gene in this missing region, *LOC124900631*. Unsurprisingly, *LOC124900631* is a close paralogue of *GPRIN2*. This novel T2T-CHM13 *GPRIN2* gene is referred to as *GPRIN2L* in this paper.

Although *GPRIN2* has been used as an illustrative example of copy number variation (CNV) in several recent publications (Handsaker *et al.* 2015, Vollger *et al.* 2022, Liao *et al.* 2023), this is an error caused by a gap in the GRCh38 assembly. The T2T-CHM13 assembly shows that this extra copy of the *GPRIN2* gene had been missing from the GRCh38 assembly. The detection of a *GPRIN2* paralogue (*GPRIN2L*) is all the more interesting because we find that the new gene is likely to be the functionally important paralogue, as was the case with WASH1 gene *LOC124908094*.

The protein sequences of the APPRIS principal isoforms (Rodriguez *et al.* 2022) of *SYT15* and *SYT15B*, and *NPY4R and NPY4R2*, are identical. However, analysis of the APPRIS principal isoforms of *GPRIN2* and *GPRIN2L* (ENSP00000363433.4 and XP_047301623.1) finds eight single amino acid differences between the two proteins as well as a single insertion of three amino acids in the *GPRIN2L* protein (Fig. 4B)*.* From the sequence evidence alone, it is not clear which sequence is closer to the ancestral gene. ENSP00000363433.4 (*GPRIN2*) has four single amino acid differences with respect to conserved mammalian *GPRIN2* sequences, while XP_047301623.1 (*GPRIN2L*) differs by three amino acids and a 3-residue insert.

However, the two genes are distinguished by the peptide evidence. ENSP00000363433.4 has no unique peptide support in PeptideAtlas, while the *GPRIN2L* protein is supported by six unique peptides with a total of 43 observations (Fig. 4B). The *GPRIN2L* protein, including the insertion, is also supported by a cDNA sequence (Nagase *et al.* 1998), a clone derived from a brain cDNA library. These results suggest that the T2T-CHM13 paralogue, *GPRIN2L*, has more functional relevance, although in this case there is nothing to suggest that *GPRIN2* is not also protein coding.

## 4 Discussion

There are many novel duplicated genes in the T2T-CHM13 human genome reference, and more than 100 have a parent gene that it is coding. However, the functional relevance of most of these duplicated genes is still unclear. The majority appear to be recent duplications, and many are likely to be pseudogenes, or at least to be in the process of pseudogenization (Lynch and Conery 2000, Wagner 2001). Here, we find that at least two of the newly uncovered duplicated genes, paralogues of *WASHC1* and *GPRIN2*, are clearly functionally important.

We have shown that one of the four novel WASH1 genes added as part of the T2T-CHM13 assembly, *LOC124908094*, produces the WASH1 protein that forms an integral part of the WASH complex, a vital complex involved in the regulation of branched actin in endosomes. Previously, the WASH1 protein was thought to be coded by *WASHC1*.

The protein produced by *LOC124908094*, WASH1-20p13, captures almost all the known peptide evidence. Seventeen of the peptides detected for WASH1 map exclusively to WASH1-20p13. WASH1-20p13 is also the human WASH1 protein that is most similar to the ancestral *WASHC1* proteins, and only differs from the predicted protein sequence produced by the GenBank *WASHC1* cDNA by one amino acid residue. We have shown that this change, G343S, is a common variant (Liao *et al.* 2023).

The other 12 WASH1 paralogues either have premature stop codons or produce a series of radical amino acid changes in conserved residues. Most are already annotated as pseudogenes. *WASHC1* on chromosome 9 is annotated as coding and produces a full-length protein. However, even though *WASHC1* does not have a disabling mutation, it would have multiple radical amino acid changes relative to WASH1-20p13 in regions that are highly conserved across vertebrates. The *WASHC1* protein is not uniquely identified by any peptide in PeptideAtlas.

Phylogenetic analysis, cross species conservation and gene neighbourhoods all suggest that these 12 WASH1 paralogues appeared in the human lineage. Alignments suggest that the gene duplications occurred after the ancestral paralogue gene, *WASH8P* on chromosome 12 (Linardopoulou *et al.* 2007), had already undergone substantial changes. If 12 WASH1 paralogues derived from a gene that was in the process of becoming a pseudogene, they too are likely to be pseudogenes.

Currently, all functional information for the WASH1 protein links to *WASHC1*. It will be important to transfer all the functional information related to the WASH1 protein from *WASHC1* to *LOC124908094*, especially since *WASHC1* is likely to be a pseudogene. It is known that the WASH complex has a role in developmental and neurodegenerative disorders (Valdmanis *et al.* 2007, Courtland *et al.* 2021), so annotating the correct WASH1 coding gene may allow the discovery of previously hidden disease-relevant variants.

As part of our analysis, we were also able to show that a novel *GPRIN2* paralogue, *GPRIN2L*, has uniquely mapped peptides in PeptideAtlas and is likely to have more functional relevance than its paralogue from the GRCh38 assembly, *GPRIN2*. Annotating the correct *GPRIN2* may help elucidate its role in neurite development.

Uncovering both the WASH1 coding gene and an important novel *GPRIN2* paralogue in the novel T2T-CHM13 assembly demonstrates the importance of annotating the missing regions in the human genome and suggests that there might be other interesting coding genes yet to be discovered in the novel regions annotated by the T2T consortium. The T2T-CHM13 assembly is a more complete and accurate reference for variant calling than the GRCh38 assembly (Aganezov *et al.* 2022). The discovery of functionally relevant coding genes in the T2T-CHM13 assembly is further confirmation of the importance of this reference set for evolutionary and biomedical research.

## Supplementary Material

vbae029_Supplementary_Data

## Data Availability

No new data or software were generated or analysed in support of this research.
