# Supplementary material for: The T2T-CHM13 reference assembly uncovers essential WASH1 and GPRIN2 paralogues
Source: Bioinform Adv. 2024 Feb 28;4(1):vbae029. doi: 10.1093/bioadv/vbae029 (PMC10924726; doi:10.1093/bioadv/vbae029)
Supplement: vbae029_Supplementary_Data [file vbae029_supplementary_data.zip › SupplementaryS2.pdf]

```
WASH4_HUMAN         MSGVMCLKASDTWASGIRSQPQGCLGKWRSMRCKHTRMHLAHLGNSRQLISLGPPRTRED
WASH6_HUMAN         -------MAFHEMQAHKNAL--------GTSGEQQAADITGPTPHQG-------------
WASH1-20q13.33      ---MTPVRMQHSLAGQTYAVPFIQPDLRREEAVQQMVDALQYLQKVS-------------
WASH1_HUMAN         ---MTPVRMQHSLAGQTYAVPFIQPDLRREEAVQQMADALQYLQKVS-------------
Gorilla_6           ---MTPLRMQHSLAGQTYAVPLIQPDLRREEAVQQMADALQYLQKVS-------------
WASH1-20p13         ---MTPVRMQHSLAGQTYAVPLIQPDLRREEAVQQMADALQYLQKVS-------------
Pygmy_12            ---MTPVRMQHSLAGQTYAVPLIQPDLRREEAVQQMADALQYLQKVS-------------
Pygmy_20            ---MTPVRMQHSLAGQTYAVPLIQPDLRREEAVQQMADALQYLQKVS-------------
Chimp_12            ---MTPVRMQHSLAGQTYAVPLIQPDLRREEAVQQMADALQYLQKVS-------------
Chimp_20            ---MTPVRMQHSLAGQTYAVPLIQPDLRREEAVQQMADALQYLQKVS-------------
                                  .   :              ::         :               

WASH4_HUMAN         G---SRISQQVEQSRSQVQAIGEKVSLAQAKIEKIKGSKKAIKVFSSAKYPAPERLQEYG
WASH6_HUMAN         G------WKQVEQSRSQVQAIGEKVSLAQAKIEKIKGSKKAIKVFSSAKYPAPERLQEYG
WASH1-20q13.33      GDIFSRISQQVEQSQSQVQAIGEKVSLAQAKIEKIKGSKKAIKVFSSAKYPAPERLQEYG
WASH1_HUMAN         GDIFSRISQQVEQSRSQVQAIGEKVSLAQAKIEKIKGSKKAIKVFSSAKYPAPGRLQEYG
Gorilla_6           GDIFSRISQRVEQSRSQVQAIGEKVSLAQAKIEKIKGSKKAIKVFSSAKYPAPERLQEYG
WASH1-20p13         GDIFSRISQRVEQSRSQVQAIGEKVSLAQAKIEKIKGSKKAIKVFSSAKYPAPERLQEYG
Pygmy_12            GDIFGRISQRVEQSRSQVQAIGEKVSLAQAKIEKIKGSKKAIKVFSSAKYPAPERLQEYG
Pygmy_20            GDIFSRISQRVEQSRSQVQAIGEKVSLAQAKIEKIKGSKKAIKVFSSAKYPAPERLQEYG
Chimp_12            GDIFGRISQRVEQSRSQVQAIGEKVSLAQAKIEKIKGSKKAIKVFSSAKYPAPERLQEYG
Chimp_20            GDIFGRISQRVEQSRSQVQAIGEKVSLAQAKIEKIKGSKKAIKVFSSAKYPAPERLQEYG
                    *       :.****.************************************** .*****

WASH4_HUMAN         SIFTDAQDPGLQRRPRHRIQSKQRPLDERAL---QEKLKDFPVCVSTKPEPEDDAEEGLG
WASH6_HUMAN         SIFTGAQDPGLQRRPRHRIQSKHRPLDERAL---QEKLKDFPVCVSTKPEPEDDAEEGLG
WASH1-20q13.33      SIFTSAQDPGLQRRPRHRIQSKHRPLDERALQPSQEKLKDFPVCVSTKPEPEDDAEEGLG
WASH1_HUMAN         SIFTGAQDPGLQRRPRHRIQSKHRPLDERAL---QEKLKDFPVCVSTKPEPEDDAEEGLG
Gorilla_6           SIFTGAQDPGLQRRSRHRIQSKHRPLDERAL---QEKLKYFPVCVSTKPEPEDDAEEGLG
WASH1-20p13         SIFTGAQDPGLQRRSRHRIQSKHRPLDERAL---QEKLKYFPVCVSTKPEPEDDAEEGLG
Pygmy_12            SIFTGAQDPGLQRHPRHRIQSKHRPLDERAL---QEKLKYFPVCVSTKPGPEDDAEEGLG
Pygmy_20            SIFTGAQDPGLQRRPRHRIQSKHRPLDERAL---QEKLKYFPVCVSTKPGPEDDAEEGLG
Chimp_12            SIFMGAQDPGLQRRPRHRIQSKHRPLDERAL---QEKLKYFPVCVSTKPGPEDDAEEGLG
Chimp_20            SIFTGAQDPGLQRRPRHRIQSKHRPLDERAL---QEKLKYFPVCVSTKPGPEDDAEEGLG
                    *** .********..*******:********   **  * ********* **********

WASH4_HUMAN         GLPSNISSVSSLLLFNTTENLYKKYVFLDPLAGAVTKTHVMLGAETEEKLFDAPLSISKR
WASH6_HUMAN         GLPSNISSVSSLLLFNTTENLYKKYVFLDPLAGAVTKTHVMLGAETEEKLFDAPLSISKR
WASH1-20q13.33      GLPSNISSVSSLLLFNTTENLYKKYVFLDPLAGAVTKTHVMLGAETEEKLFDAPLSISKR
WASH1_HUMAN         GLPSNISSVSSLLLFNTTENLYKKYVFLDPLAGAVTKTHVMLGAETEEKLFDAPLSISKR
Gorilla_6           GLPSNISSVSSLLLFNTTENLYKKYVFLDPLAGAVTKTHVMLGAETEEKLFDAPLSISKR
WASH1-20p13         GLPSNISSVSSLLLFNTTENLYKKYVFLDPLAGAVTKTHVMLGAETEEKLFDAPLSISKR
Pygmy_12            GLPSNISSVSSLLLFNTTENLYKKYVFLDPLAGAVTKTRVMLGAETEEKLFDAPLSISKR
Pygmy_20            GLPSNISSVSSLLLFNTTENLYKKYVFLDPLAGAVTKTHVMLGAETEEKLFDAPLSISKR
Chimp_12            GLPSNISSVSSLLLFNTTENLYKKYVFLDPLAGAVTKTHVMLGAETEEKLFDAPLSISKR
Chimp_20            GLPSNISSVSSLLLFNTTENLYKKYVFLDPLAGAVTKTHVMLGAETEEKLFDAPLSISKR
                    ********************* ****************.*********************

WASH4_HUMAN         EQLEQQVPENYFYVPDLGQVPEIDVPSYLPDLPGIANDLMYIADLGPGIAPSAPGTIPEL
WASH6_HUMAN         EQLEQQVPENYFYVPDLGQVPEIDVPSYLPDLPSIANDLMYSADLGPGIAPSAPGTIPEL
WASH1-20q13.33      EQLEQQVPENYFYVPDLGQVPEIDVPSYLPDLSGIANDLMYIADLGPGIAPSAPGTIPEL
WASH1_HUMAN         EQLEQQVPENYFYVPDLGQVPEIHVPSYLPDLPGIANDLMYSADLGPGIAPSAPGTIPEL
Gorilla_6           EQLEQQVPENYFYVPDLGQVPEIDVPSYLPDLPGIANDLMYSVDLGPGIAPSAPGTIPEL
WASH1-20p13         EQLEQQVPENYFYVPDLGQVPEIDVPSYLPDLPGIANDLMYSADLGPGIAPSAPGTIPEL
Pygmy_12            EQLEQQVPENYFYVPDLGQVPEIDVPSYLPDLPGIANDLMYSADLGPGIAPSAPGTIPEL
Pygmy_20            EQLEQQVPENYFYVPDLGQVPEIDVPSYLPDLPGIANDLMYSADLGPGIAPSAPGTIPEL
Chimp_12            EQLEQQVPENYFYVPDLGQVPEIDVPSYLPDLPGIANDLMYSADLGPGIAPSAPGTIPEL
Chimp_20            EQLEQQVPENYFYVPDLGQVPEIDVPSYLPDLPGIANDLMYSADLGPGIAPSAPGTIPEL
                    *********************** ********..*:*****  .*****************

WASH4_HUMAN         PTFHTEVAEPLKVDLQDGVLT---PPPPPPPPPPAPEVLASAPPLPPSTAAPVGQGARQD
WASH6_HUMAN         PTFHTEVAEPLKADLQDGVLT---PPPPPPPPPPAPEVLASAPPLPPSTAAPVGQGARQD
WASH1-20q13.33      PTFHTEVAEPLKVDLQDGVLT---PPPPPPPPPPAPEVLASAPPLPPSTAAPVGQGARQD
WASH1_HUMAN         PTFHTEVAEPLKVDLQDGVLT---PPPPPPPPPPAPEVLASAPPLPPSTAAPVGQGARQD
Gorilla_6           PTFHTEVAEPLKADLQDGVLTAPPPPPPPPPPPPPAPEVLASAPPLPPSTAAPVGQGARQD
WASH1-20p13         PTFHTEVAEPLKADLQDGVLTAPPPPPPPPPPPPPAPEVLASASPLPPSTAAPVGQGARQD
Pygmy_12            PTFHTEVAEPLKADLQDGVLTAPPPPPPPPPPPPPAPEVLASAPPLPPSTVAPVGQGARQD
Pygmy_20            PTFHTEVAEPLKADLQDGVLTAPPPPPPPPPPPPPAPEVLASAPPLPPSTAAPVGQGARQD
```

```
Chimp_12                PTFHTEVAEPLKADLQDGVLTAPPPPPPPPPPPPPAPEVLASAPPLPPSTAAPVGQGARQD
Chimp_20                PTFHTEVAEPLKADLQDGVLTAPPPPPPPPPPPPPAPEVLASAPPLPPSTAAPVGQGARQD
                        ************.********   ******************.******.**********

WASH4_HUMAN             DSSSSASPSVQGAPREVVDPSGGWATLLESIRQAGGIGKAKLRSMKERKLEKQQQKEQEQ
WASH6_HUMAN             DGSSSASPSVQGAPREVVDPSGGWATLLESIRQAGGIGKAKLRSMKERKLEKKKQKEQEQ
WASH1-20q13.33          DSSSSASPSVQGAPREVVDPSGGWATLLESIRQAGGISKAKLRSMKERKLEKKQQKEQEQ
WASH1_HUMAN             DSSSSASPSVQGAPREVVDPSGGWATLLESIRQAGGIGKAKLRSMKERKLEKQQQKEQEQ
Gorilla_6               DSSSIASPSVQGAPREVVDPSGGRATLLESIRQAGGIGKAKLRSVKERKLEKKKQKEQEQ
WASH1-20p13             DGSSSASPSVQGAPREVVDPSGGRATLLESIRQAGGIGKAKLRSVKERKLEKKKQKEQEQ
Pygmy_12                DSSSSASPSVQGAPREVVDPSGGRATLLESIRQAGGIGKAKLRSVKERKLEKKQQKEQEQ
Pygmy_20                DSSSSASPSVQGAPREVVDPSGGRATLLESIRQAGGIGKAKLRSVKERKLEKKKQKEQEQ
Chimp_12                DSSSSASPSVQGAPREVVDPSGGRATLLESIRQAGGIGKAKLRSVKERKLEKKKQKEQEQ
Chimp_20                DSSSSASPSVQGAPREVVDPSGGRATLLESIRQAGGIGKAKLRSVKERKLEKKKQKEQEQ
                        *.** :*****************.*************.******:*******::******

WASH4_HUMAN             VRATSQGGHLMSDLFNKLVMRRKGISGKGPGAGDGPGGAFARVSDSIPPLPPPQQPQA-E
WASH6_HUMAN             VRATSQGGHLMSDLFNKLVMRRKGISGKGPGAGEGPGGAFARVSDSIPPVPPPQQPQAEE
WASH1-20q13.33          VRATSQGGHLMSDLFNKLVMRRKGISGKGPGAGEGPGGAFARVSDSIPPLPPPQQPQAEE
WASH1_HUMAN             VRATSQGGHLMSDLFNKLVMRRKGISGKGPGAGEGPGGAFVRVSDSIPPLPPPQQPQAEE
Gorilla_6               VRATSQGGDLMSDLFNKLVMRRKGISGKGPGAGEGPGGAFARVSDSIPPLPPPQQPQAEE
WASH1-20p13             VRATSQGGDLMSDLFNKLVMRRKGISGKGPGAGEGPGGAFARVSDSIPPLPPPQQPQAEE
Pygmy_12                VRATSQGGDLMSDLFNKLVMRRKGISGKGPGAGEGPGGAFARVSDSIPPLPPPQQPQAEE
Pygmy_20                VRATSQGGDLMSDLFNKLVMRRKGISGKGPGAGEGPGGAFARVSDSIPPLPPPQQPQAEE
Chimp_12                VRATSQGGDLMSDLFNKLVMRRKGISGKGPGAGEGPGGAFARVSDSIPPLPPPQQPQAEE
Chimp_20                VRATSQGGDLMSDLFNKLVMRRKGISGKGPGAGEGPGGAFARVSDSIPPLPPPQQPQAEE
                        ******** ************.***********:******.********:*******. *

WASH4_HUMAN             DEDDWES
WASH6_HUMAN             DEDDWES
WASH1-20q13.33          DEDDWES
WASH1_HUMAN             DEDDWES
Gorilla_6               DEDDWES
WASH1-20p13             DEDDWES
Pygmy_12                DEDDWES
Pygmy_20                DEDDWES
Chimp_12                DEDDWES
Chimp_20                DEDDWES
                        *:*****
```

Supplementary figure 2

Alignments of predicted *WASHC1* sequences annotated in chimpanzee, bonobo (pygmy chimpanzee) and gorilla with the five intact WASH1 genes annotated in the CHM13 assembly (those without disabling mutations). Where the human WASH1 proteins differ in sequence is highlighted in red.
