# Supplementary material for: The T2T-CHM13 reference assembly uncovers essential WASH1 and GPRIN2 paralogues
Source: Bioinform Adv. 2024 Feb 28;4(1):vbae029. doi: 10.1093/bioadv/vbae029 (PMC10924726; doi:10.1093/bioadv/vbae029)
Supplement: vbae029_Supplementary_Data [file vbae029_supplementary_data.zip › SupplementaryS1.pdf]

```
Zebrafish                  ---MVR-MTQKRYLEGQVYSVPLIQPDLRREEAVHQITDALQYLEMISTDIF--------
Mexican cavefish           ---MVR-MTQKYHLEGQVYSVPVIQQDLRREEAVHQITDALQYLENISTDIF--------
Electric eel               ---FSPLMTQKHHLEGQVYSVPLIQQDLRREEAVHQITDALLYLENISTDIF--------
African clawed frog        -------MPQNRSVESQAYSLPLILPDLRREEAIHQITDTLQHLQTVSNDIF--------
Western clawed frog        -------MPQNRSMESQAYSLPLILPDLRREEAIHQITDTLQHLQTVSNDIF--------
Microcaecilia unicolor     -------MPQKNIVPGQTYSVPLIQPDLRREEAIHQIADALQYLQQVSSDIF--------
Chinese alligator          ---MAT-VTQKHFLEGQTYSVPLIQPDLRREEAILQVADALQYLQKVSGDIF--------
Saltwater crocodile        ---MAT-VTQKHFLEGQTYSVPLIQPDLRREEAILQVVDALQYLQKVSGDIF--------
Chicken                    ---MTT-VAQKHFLEGQTYSVPLIQPDLRREEAVQQVADALQYLQKVSGDIF--------
Kakapo                     ---MTT-VAQKHFLEGQTYSVPLIQPDLRREEAVQQVADALQYLQKVSGDIF--------
Degu                       ---MTT-VRMQHSLAGQTYAVPLIQPDLRREEAIQQVADALQYLQEVSRDIF--------
Tasmanian devil            ---MTT-MAQSHYLEGQAYSVPLIQPDLRREEAIQQVADALQYLQKVSGDIF--------
Koala                      ---MTT-MMQSHYLEGQAYSVPLIQPDLRREEAIQQVADALQYLQKVSGDIF--------
Golden hamster             ---MTP-LKTQHSLVGQPYAVPLIQPDLRREEAIQQVADALQHLQSISGDIF--------
Rat                        ---MTA-VKTQHSLAGQVYAVPLIQPDLRREEAIQQVADALQYLQNISGDIF--------
Mouse                      ---MTP-VKTQCSLAGQLYAVPLIQPDLRREEAIQQVADALQYLQNISGDIF--------
Blue whale                 ---MTP-TGTQRPLADQTYAVPLIQPDLRREEATQQVADALQHLQKVSGDIF--------
American beaver            ---MTP-VRTQHSLAGQTYAVPLIQPDLRREEAIQQVADALQYLQKVSGDIF--------
Pig                        ---MTP-TGTQHSLAGQTYTVPLIQPDLRREEAIQQVADALQYLQKVSGDIF--------
Panther                    ---MTP-MRTQHSLAGQTYAVPLIQPDLRREEAIQQVADALQYLQKVSGDIF--------
Cheetah                    ---MTP-MRTQHSLAGQTYAVPLIQPDLRREEAIQQVADALQYLQKVSGDIF--------
Dog                        ---MTP-MRTQHSLAGQTYAVPLIQPDLRREEAIQQMADALQYLQKVSGDIF--------
Monk seal                  ---MTP-TRTQHSLAGQTYAVPLIQPDLRREEAIQQVADALQYLQKVSGDIF--------
Polar bear                 ---MTP-TRSQHSLAGQTYAVPLIQPDLRREEAIQQAADALQYLQKVSGDIF--------
European ferret            ---MTP-TRTQHSLAGQTYAVPLIQPDLRREEAIQQVADALQYLQKVSGDIF--------
Ugandan colobus            ---MTP-VRTQHSLAGQTYAVPLIQPDLRREEAVQQMADALQYLQKVSGDIF--------
Chimpanzee Chr20           ---MTP-VRMQHSLAGQTYAVPLIQPDLRREEAVQQMADALQYLQKVSGDIF--------
WASH1-20p13                ---MTP-VRMQHSLAGQTYAVPLIQPDLRREEAVQQMADALQYLQKVSGDIF--------
WASHC1                     ---MTP-VRMQHSLAGQTYAVPFIQPDLRREEAVQQMADALQYLQKVSGDIF--------
WASHP4                     MSGVMC-LKASDTWASGIRSQPQGCLGKWRSMRCKHTRMHLAHLGNSRQLISLGPPRTRE
WASHP6                     --------MAFHEMQAHKNAL--------GTSGEQQAADITGPTPHQGG-----------
WASHP3                     ---MTP-VRMQHSLAGQTYAVPLIQPDLRREEAVQQMADALQYLQKVSGDIF--------
WASHP2                     ---MTP-VRMQHSLAGQTYAVPLIQPDLRX------------------------------
                                              :               :

Zebrafish                  --TRVSESVEKNRAHLQSVTDRIKLAQARVQKIKGSKKATKVFSSAKYPAPEKLQDYSSI
Mexican cavefish           --TRVSQSVEKNRTYLQAVTDRIKLAQARVHKIKGSKKATKVFSSAKYPAPEKLEDYSSI
Electric eel               --NRVSQSVERNRSHLQAVADRIKLAQARVQKIKGSKKATKVFSSAKYPAPEKLQDYSSI
African clawed frog        --SRILQRVETNRDQLQRINGRLSLAQAKIERLKGSKKAIKVFSSAKYPAPDRLQEYSSI
Western clawed frog        --SRILQRVETNRGQLQRINGRLSLAQAKIERLKGIKKAIKVFSSAKYPAPEHLQEYSSV
Microcaecilia unicolor     --NRISQRVETSRTQLQLINERVALAHAKIKKIQGSKKAIKVFSSAKYPAQDKLQEYSSI
Chinese alligator          --NRISQRVEASRMQLQAIGERVTLAQAKIEKIKGSKKAIKVFSSAKYPAPERLQEYSSI
Saltwater crocodile        --NRISQRVEASRTQLQAIGERVTLAQAKIEKIKGSKKAIKVFSSAKYPAPERLQEYSSI
Chicken                    --NRISQRVETSRAQLQAISERVTLAQAKIEKIKGSKKAIKVFSSAKYPAPERLQEYCSI
Kakapo                     --NRISQRVEASRAQLQAIGERVTLAQAKIEKIKGSKKAIKVFSSAKYPAPERLQEYSSI
Degu                       --NRISQRVELSLGQLQVLGERISASQAKIQRIKGSKKAIKVFSSAKYPAPERLREHSSI
Tasmanian devil            --SRISQRVEMSRSQLQVIGERVSLAQAKIEKIKGSKKAIKVFSSAKYPAPDRLQEYNSI
Koala                      --SRISQRVEMSRSQLQVIGQRVSLAQAKIEKIKGSKKAIKVFSSAKYPAPERLQEYNSI
Golden hamster             --SRISQRVELSRRQLQAIRERVSLAQAKIEKIKGSKKAIKVFSSAKYPAPERLQEYGSI
Rat                        --SRISQRVELSRRQLQAIGERVSLAQAKIEKIKGSKKAIKVFSSAKYPAPEHLQEYNSV
Mouse                      --SRISRRVELSRRQLQAISERVSLAQAKIEKIKGSKKAIKVFSSAKYPAPEHLQEYGSI
Blue whale                 --SRISQRVELSRSQLQAIGERVSLAQAKIEKIKGSKKAIKVFSSAKYPAPERLQEYSSI
American beaver            --SRISQRVELSRSQLQAIGERVSLAQAKIEKIKGSKKAIKVFSSAKYPAPEHLQEYGSI
Pig                        --SRISQRVELSRRQLQAIGERVSLAQAKIEKIKGSKKAIKVFSSAKYPAPERLREYGSI
Panther                    --SRISQRVELSRRQLQAIGERVSLAQAKIEKIKGSKKAIKVFSSAKYPAPERLQEYGSI
Cheetah                    --SRISQRVEFSRRQLQAIGERVSLAQAKIEKIKGSKKAIKVFSSAKYPAPERLQEYSSI
Dog                        --SRISQRVELSRKQLQAIGERVSLAQAKIEKIKGSKKAIKVFSSAKYPAPERLQEYDSI
Monk seal                  --SRISQRVELSRKQLQAIGERVSLAQAKIEKIKGSKKAIKVFSSAKYPAPERLQEYGSI
Polar bear                 --SRISQRVELSRRQLQAIGERVSLAQAKIEKIKGSKKAIKVFSSAKYPAPERLQEYGSI
European ferret            --SRISQRVELSRRQLQAIGERVSLAQAKIEKIKGSKKAIKVFSSAKYPAPERLQEYGSI
Ugandan colobus            --SRISQRVEQSRSQVQAIGEKVSLAQAKIEKIKGSKKAIKVFSSAKYPAPERLQEYGSI
Chimpanzee Chr20           --GRISQRVEQSRSQVQAIGEKVSLAQAKIEKIKGSKKAIKVFSSAKYPAPERLQEYGSI
WASH1-20p13                --SRISQRVEQSRSQVQAIGEKVSLAQAKIEKIKGSKKAIKVFSSAKYPAPERLQEYGSI
WASHC1                     --SRISQQVEQSRSQVQAIGEKVSLAQAKIEKIKGSKKAIKVFSSAKYPAPGRLQEYGSI
WASHP4                     DGSRISQQVEQSRSQVQAIGEKVSLAQAKIEKIKGSKKAIKVFSSAKYPAPERLQEYGSI
WASHP6                     -----WKQVEQSRSQVQAIGEKVSLAQAKIEKIKGSKKAIKVFSSAKYPAPERLQEYGSI
WASHP3                     --SRISQQVEQSRSQVQAIGEKVSLAQAKIEKIKGSKKAIKVFSSAKYPAPERLQEYGSI
                                   ** .   :* :  .:  ::*.: .::* *** **********  .* :: *:
```

```
Zebrafish                   FTGAVDPASQKRPRIKVQSKLRPLDDKAQQEKLMYLPVCVNTKKRSEDETEEGLGSLPRN
Mexican cavefish            FTGAVDPASQKRPRYKIQGKLRPLDDKALQEKLKYFPVFVNTKKRSEDETEEGLGSLPRN
Electric eel                FMGAIDPVSQKRPRYKVQAKLRPLDDKALQEKLMYFPVCVNTKKRSDDETEEGLGSLPRN
African clawed frog         FAGAKDGWSAKKQRHKIQSKHRPLDEQAVQEKLKYFPVCVNTRGQDEESAEEGLGSLPRN
Western clawed frog         FAGAEDGWLAKKLRHKIQSKHRPLDEQAVQEKLKYFPVCVNTRGQDEESAEEGLGSLPRN
Microcaecilia unicolor      FAGVENQSPQKRPRHKIQSKHRLLEEKTLQEKLKYFPVCVNTQVHEDDEAEGGLGSLPRN
Chinese alligator           FAGAEDPAKQKGQKHKVQSKHRVLDEKCLQEKLKYFPVCASTTVHQEDDAEEGLGSLPRN
Saltwater crocodile         FAGAEDPAKQKGQKHKVQSKHRVLDEKCLQEKLKYFPVCASTTVYQEDDAEEGLGSLPRN
Chicken                     FAGAEDPSKQKWPRHKIQSKHRMLDEKSLQEKLKYFPVCVNTKIHQEDDAEEGLGSLPRN
Kakapo                      FAGAEDPAKQKWPRHKIQSKHRMLDEKALQEKLKYFPVCVSTKIHQEDDAEEGLGSLPRN
Degu                        FSGAQDPGPLCPPRRRIQSKHRALDERALQEKLKYFPVCVSARPEPEDEAEEGLGGLPSN
Tasmanian devil             FEGAEDPGSQRRIRHKIQSKHRPLDERALQEKLKYFPVCVSTRPQPEDEAEEGLGGLPSN
Koala                       FKGAQDPGSQRHIRHKIQSKHRPLDERALQEKLKYFPVCVSTKPQPEDEAEEGLGGLPSN
Golden hamster              FTGALDPGLQRRPRYRIQSKHRPLDERALQEKLKYFPVCVSTKVEPEDEAEEGLGGLPSN
Rat                         FTGALDPGLQRRPRYRIQSKHRPLDERALQEKLKYFPVCVSTKSEPEDEAEEGLGGLPSN
Mouse                       FTGALDPGLQRRPRYRIQSKHRPLDERALQEKLKYFPVCVNTKSEPEDEAEEGLGGLPSN
Blue whale                  FTGAQDPGLQRRARHRVQSKHRPLDELALQEKLKYFPVCVSTKPEPEDEAEEGLGGLPSN
American beaver             FTGAQDPGLQRRPRHRIQSKHRPLDERALQEKLKYFPVCVTTKPEPEDEAEEGLGGLPSN
Pig                         FTGAQDPGLQRRPRHRIQSKHRPLDERALQEKLKYFPVCVSTKPEPEDEAEEGLGGLPSN
Panther                     FTGAQDPGVQRRPRHRIQSKHRPLDEWALQEKLKYFPVCVNTKQEPEDEAEEGLGGLPSN
Cheetah                     FTGAQDPGVQRRPRHRIQSKHRPLDEWALQEKLKYFPVCVNTKPEPEDEAEEGLGGLPSN
Dog                         FTGAQDPGLQRRPRYRIQSKHRPLDEWALQEKLKYFPVCVNTKPEPEDEAEEGLGGLPSN
Monk seal                   FTGAQDPGLQRRPRHRIQSKHRPLDEWALQEKLKYFPVCVNTKPEPEDEAEEGLGGLPSN
Polar bear                  FMGAQDPGLQRRPRHRIQSKHRPLDEWALQEKLKYFPVCVNTKPEPEDEAEEGLGGLPSN
European ferret             FTGAQDPGLQRRPRHRIQSKHRPLDEWALQEKLKYFPVCVNTKPEPEDEAEEGLGGLPSN
Ugandan colobus             FTGAQDPGLQRRPRHRIQSKHRPLDEQALQEKLKYFPVCVSAKPEPEDDAEEGLGGLPSN
Chimpanzee chr20            FTGAQDPGLQRRPRHRIQSKHRPLDERALQEKLKYFPVCVSTKPGPEDDAEEGLGGLPSN
WASH1-20p13                 FTGAQDPGLQRRSRHRIQSKHRPLDERALQEKLKYFPVCVSTKPEPEDDAEEGLGGLPSN
WASHC1                      FTGAQDPGLQRRPRHRIQSKHRPLDERALQEKLKDFPVCVSTKPEPEDDAEEGLGGLPSN
WASHP4                      FTDAQDPGLQRRPRHRIQSKQRPLDERALQEKLKDFPVCVSTKPEPEDDAEEGLGGLPSN
WASHP6                      FTGAQDPGLQRRPRHRIQSKHRPLDERALQEKLKDFPVCVSTKPEPEDDAEEGLGGLPSN
WASHP3                      FTGAQDPGLQRRPRHRIQSKHRPLDERALQEK----------------------------
                            * .. :       . .:*.* * *::   ****  :** ..:    ::.:* ***.** *

Zebrafish                   VNSVSSLLLFNTTENL-YKKYVFLDPLAGAVTKTHTTLETEKEDKPFDAPLSITKREQLE
Mexican cavefish            ISSVSSLLLFNTTENL-YKKYVFLDPLAGAVTKTHTTLETEKEEKPFDAPLSITKREQLE
Electric eel                ISSISSLLLFNTTENL-YKKYVFLDPLAGAITKTHTALETEKEEKPFDAPLSITKREQLE
African clawed frog         INSVSSLLLFNTTENL-YKKYVLLDPLAGVVTRTNPALEGEDEEKLFDAPLSITKREQLE
Western clawed frog         ISSVSSLLLFNTTENL-YKKYVFLDPLAGVVTRTNPALEGEDEEKLFDAPLSITKREQLE
Microcaecilia unicolor      INSVSSLLLFNTTENL-YKKYVLLDPLAGAVTKTHMTLETETEEKLFDAPLSISKREQLE
Chinese alligator           ISSLSSLLLFNTTENL-YKKYVFLDPLAGAVTKTHVALETEMEEKLFDAPLSITKRGQLD
Saltwater crocodile         ISSLSSLLLFNTTENL-YKKYVFLDPLAGAVTKTHVALETEMEEKLFDAPLSITKRGQLD
Chicken                     ISSLSSLLLFNTTENL-YKKYVFLDPLAGAVTKTHVALETETEEKLFDAPLSITERGQLD
Kakapo                      ISSLSSLLLFNTTENL-YKKYVFLDPLAGAVTKTHVALETETEEKLFDAPLSITKRGQLD
Degu                        ISSVSSLLLFNTTENL-YKKYVFLDPLAGAVTKTHVMLGTEKQEKLFDAPLSISKREQLA
Tasmanian devil             INSLSSLLLFNTTENL-YKKYVFLDPLAGAVTKTHVTLGTETE-KLFDAPLSISKREQLE
Koala                       INSLSSLLLFNTTENL-YKKYVFLDPLAGAVTKTHVTLGTETE-KLFDAPLSISKREQLE
Golden hamster              ISSISSLLLFNTTENL-YKKYVFLDPLAGAVTKTHTMLGTE-EEKLFDAPLSISKREQLE
Rat                         ISSISSLLLFNTTENL-YKKYVFLDPLAGAVTKTHTMLGTE-EEKLFDAPLSISKREQLE
Mouse                       ISSISSLLLFNTTENL-YKKYVFLDPLAGAVTKTHTMLGTE-EEKLFDAPLSISKREQLE
Blue whale                  ISSVSSLLLFNTTENL-YKKYVFLDPLAGAVTKTHVMLGAETEEKLFDAPLSISKREQLE
American beaver             ISSVSSLLLFNTTENL-YKKYVFLDPLAGAVTKTHVMLGADTEEKLFDAPLSISKREQLE
Pig                         ISSVSSLLLFNTTENL-YKKYVFLDPLAGAVTKTHVMLGAETEEKLFDAPLSISKREQLE
Panther                     ISSVSSLLLFNTTENL-YKKYVFLDPLAGAVTKTHVMLGAETEEKLFDAPLSISKREQLE
Cheetah                     ISSVSSLLLFNTTENL-YKKYVFLDPLAGAVTKTHVMLGAETEEKLFDAPLSISKREQLE
Dog                         ISSVSSLLLFNTTENL-YKKYVFLDPLAGAVTKTHVMLGAETEEKLFDAPLSISKREQLE
Monk seal                   ISSVSSLLLFNTTENLRYKKYVFLDPLAGAVTKTHVTLGAETEEKLFDAPLSISKRGQLE
Polar bear                  ISSVSSLLLFNTTENL-YKKYVFLDPLAGAVTKTHVMLGAETEEKLFDAPLSISKREQLE
European ferret             ISSVSSLLLFNTTENL-YKKYVFLDPLAGAVTKTHVMLGAETEEKLFDAPLSISKREQLE
Ugandan colobus             ISSVSSLLLFNTTENL-YKKYVFLDPLAGAVTKTHVMLGAETEEKLFDAPLSISKREQLE
Chimpanzee Chr20            ISSVSSLLLFNTTENL-YKKYVFLDPLAGAVTKTHVMLGAETEEKLFDAPLSISKREQLE
WASH1-20p13                 ISSVSSLLLFNTTENL-YKKYVFLDPLAGAVTKTHVMLGAETEEKLFDAPLSISKREQLE
WASHC1                      ISSVSSLLLFNTTENL-YKKYVFLDPLAGAVTKTHVMLGAETEEKLFDAPLSISKREQLE
WASHP4                      ISSVSSLLLFNTTENL-YKKYVFLDPLAGAVTKTHVMLGAETEEKLFDAPLSISKREQLE
WASHP6                      ISSVSSLLLFNTTENL-YKKYVFLDPLAGAVTKTHVMLGAETEEKLFDAPLSISKREQLE
                            :.*:************  ****:******.:*.*:  *  : : * *******::* **
```

```
Zebrafish                  RQTAENYFYVPDLGQVPEIDVPSYLPDLPGIADDLMYSADLGPGFAPSVPASNSIPELPS
Mexican cavefish           RQTAENYFYVPDLGQVPEIDVPSYLPDLPGIADDLMYSADLGPGFAPSGPASATIPELPS
Electric eel               RQTAENYFYVPDLGQVPEIDVPSYLPDLPGIADDLMYSADLGPGFAPSVPANTAIPELPS
African clawed frog        RQTAENYFYVPDLGQVPEIDVPYSLPDLLGVADDLMYSADLGPGIAPSAP-GVPIPELPT
Western clawed frog        RQTAENYFYVPDLGQVPEIDVPYSLPDLPGVADDLMYSADLGPGIAPSAP-GVPIPELPT
Microcaecilia unicolor     RQTAENYFYVPDLGLVPEIDVPAYLPDLPGIADDLMYCADLGPGIAPSAPSG-TIPELPT
Chinese alligator          QQVAENYFYVPDLGQVPEIDVPSYLPDLPGIAADLMYSADLGPGIAPSAP-SSAIPELPT
Saltwater crocodile        QQVAENYFYVPDLGQVPEIDVPSYLPDLPGIAADLMYSADLGPGIAPSAP-SSAIPELPT
Chicken                    RQVAENYFYVPDLGQVPEIDVPSYLPDLPGIAADLMYSADLGPGIAPSAP-SSAIPELPT
Kakapo                     RQVAENYFYVPDLGQVPEIDVPSYLPDLPGIAADLMYSADLGPGIAPSAP-SSAIPELPT
Degu                       QQVPENYFYVPDLGQVPEIDVPSYLPDLPGIADDLMYSADLGPGIAPSAP-G-AIPELPT
Tasmanian devil            QQVAENYFYVPDLGQVPEIDVPSYLPDLPGIADDLMYSADLGPGIAPSAP-G-TIPELPT
Koala                      QQVAENYFYVPDLGQVPEIDVPSYLPDLPGIADDLMYSADLGPGIAPSAP-G-TIPELPT
Golden hamster             QQAPENYFYVPDLGQVPEIDVPSYLPDLPGIADDLMYSADLGPGIAPSAP-G-AIPELPT
Rat                        QPAPENYFYVPGLGQVPEIDVPSYLPDLPGVADDLMYSADLGPGIAPSAP-G-AIPELPA
Mouse                      RQAPENYFYVPDLGQVPEIDVPSYLPDLPGVADDLMYSADLGPGIAPSAP-G-AIPELPA
Blue whale                 RQVPENYFYVPDLGQVPEIDVPSYLPDLPGVADDLMYSADLGPGIAPSAP-G-TIPELPA
American beaver            RQIPENYFYVPDLGQVPEIDVPSYLPDLPGIADDLMYSADLGPGIAPSAP-G-AIPELPT
Pig                        QQMPENYFYVPDLGQVPEIDVPSYLPDLPGVADDLMYSADLGPGIAPSAP-G-TIPELPT
Panther                    QQVPENYFYVPDLGQVPEIDVPSYLPDLPGIADDLMYSADLGPGIAPSAP-G-TIPELPT
Cheetah                    QQVPENYFYVPDLGQVPEIDVPSYLPDLPGIADDLMYSADLGPGIAPSAP-G-TIPELPT
Dog                        QQVPENYFYVPDLGQVPEIDVPSYLPDLPGIADDLMYSADLGPGIAPSAP-G-TIPELPT
Monk seal                  QQVPENYFYVPDLGQVPEIDVPSYLPDLPGIADDLMYSADLGPGIAPSAP-G-TIPELPA
Polar bear                 QQVPENYFYVPDLGQVPEIDVPSYLPDLPGIADDLMYSADLGPGIAPSAP-G-TIPELPT
European ferret            QQVPENYFYVPDLGQVPEIDVPSYLPDLPGIADDLMYSADLGPGIAPSAP-G-TIPELPT
Ugandan colobus            QQVPENYFYVPDLGQVPEIDVPSYLPDLPGIANDLMYSADLGPGIAPSAP-G-TIPELPT
Chimpanzee Chr20           QQVPENYFYVPDLGQVPEIDVPSYLPDLPGIANDLMYSADLGPGIAPSAP-G-TIPELPT
WASH1-20p13                QQVPENYFYVPDLGQVPEIDVPSYLPDLPGIANDLMYSADLGPGIAPSAP-G-TIPELPT
WASHC1                     QQVPENYFYVPDLGQVPEIHVPSYLPDLPGIANDLMYSADLGPGIAPSAP-G-TIPELPT
WASH4                      QQVPENYFYVPDLGQVPEIDVPSYLPDLPGIANDLMYIADLGPGIAPSAP-G-TIPELPT
WASH6                      QQVPENYFYVPDLGQVPEIDVPSYLPDLPSIANDLMYSADLGPGIAPSAP-G-TIPELPT
                           .  .*******.** **** **  **** .:: **** ******:*** * . .*****:

Zebrafish                  FGTD-HDESSGSDSQFKLEAP--PPPPPPPPPPPEPTHVPVPPPGTSAAPPPP--P----
Mexican cavefish           FGTD-HEEPNGPGSQFRPEAPP-PPPPPPPPPPPEPTHVPGPPPGAPPAPPPP--P-PPP
Electric eel               FGTD-HEEPNGHGESQAKLFAPPPPPPPPPPPPPPPP-----PPPGTPPAPPPP----PPL
African clawed frog        FTTEDITENSITDRQDGRLLPP-PPPPPPPPPPPP-----PEPSALS-PPAP--P-PPP
Western clawed frog        FITEDITENSRTDSQDGRLLPP-PPPPPPPPPPPP-----PEPSVLS-PPTS--L-APP
Microcaecilia unicolor     FNIE-SLDIFKPDLRDEGMMLS-LSPPPPPPPPPPP-----PVITSHI-PPPP--P-PPP
Chinese alligator          FSTE-SAGAFQPGLPDGVLM---PPPPPPPPPPPP-----PVMPATA-PPPPPLP-QPA
Saltwater crocodile        FSTE-STGAFQPDLQDGVLM---PPPPPPPPPPPP-----PVMPATA-PPPPPLP-QPT
Chicken                    FSTE-SVEPSQADLQDPGLL-----PPPPPPPPPPP-----PVMPTTV-PPPPPLP-QPT
Kakapo                     FNTE-SVERLQPDLQDAELM----PPPPPPPPPPPP-----PVMPSTV-PPPPPLP-QPT
Degu                       FQTE-VAEPFKPDPEGVVLPAP-PPPPPPPPPPPA-----PAVLASAPPGLP--P-QTA
Tasmanian devil            FNTE-LAEPFKPELDGDTPVAP-PPPPPPPPPPPA-----PAVLVSAPPPPP--P-PQL
Koala                      FNTE-LAEPFKPELHGETPIAP-PPPPPPPPPPPA-----PAVLVSA-PPPP--P-LPQ
Golden hamster             FHTE-VAEPFQPELDGGVLLAA-PPPPPPPPPPPA-----PAVVVST-PQPPVFP-EVA
Rat                        FHTE-VAEPFQPEREDGALLAPPPPPPPPPPPPPPA-----PTAVVSA-PQPPMSP-DVV
Mouse                      FHTE-VAEPLQPELENEVLLAAPPPPPPPPPPPPPA-----PTALVST-PQPPMFP-DMA
Blue whale                 FHTE-VAEPFKPE--DGVLAV----PPPPPPPPPPA-----PALLVSALPPPP--PAQTV
American beaver            FHTE-IAEPFKPDLEDGVLAA--PPPPPPPPPPLA-----PAVLTSAPPPPP--P-QTT
Pig                        FHTE-VAEPFKPDLEDGVLTA---PPPPPPPPPPPA-----PAVLVSA-PPPP--P-PPT
Panther                    FHTE-VAEPFKPDPEDGVLIA--PPPPPPPPPPPA-----PAVLVSA-PPPP--P-QTL
Cheetah                    FHTE-VAEPFKPDPEDGVLIA--PPPPPPPPPPPA-----PAVLVSA-PPPP--P-QTL
Dog                        FHTE-VAETFKPDLEDGVLTA--PPPPPPPPPPPA-----PAVLVSA-PPPP--P-QAI
Monk seal                  FHTE-VAETFRPDPEDGVLTA--PPPPPPPPPPPA-----PAVLVSA-PPPP--P-QPV
Polar bear                 FHTE-VAETFKPDPEDGVLTA--PPPPPPPPPPPA-----PAVLVSA-PPPP--P-QPM
European ferret            FHTE-VAETFKPDPEDGVLTA--PPPPPPPPPPPA-----PAVLVNA-PPPL--P-QPM
Ugandan colobus            FHTE-VAEPLKTDLEDGVLAA--PPPPPPPPPPPA-----PEVLASA-PPLP--P-STA
Chimpanzee Chr20           FHTE-VAEPLKADLQDGVLTA--PPPPPPPPPPPA-----PEVLASA-PPLP--P-STA
WASH1-20p13                FHTE-VAEPLKADLQDGVLTA--PPPPPPPPPPPA-----PEVLASA-SPLP--P-STA
WASHC1                     FHTE-VAEPLKVDLQDGVLT-----PPPPPPPPPPA-----PEVLASA-PPLP--P-STA
WASHP4                     FHTE-VAEPLKVDLQDGVLT-----PPPPPPPPPPA-----PEVLASA-PPLP--P-STA
WASHP6                     FHTE-VAEPLKADLQDGVLT-----PPPPPPPPPPA-----PEVLASA-PPLP--P-STA
                           *  :                     ********* .     *       .
```

```
Zebrafish                     PPPPMTADNTDASSPAPPTGTVKGAPSEVVQPSNGRASLLESIRNAGGIGKANLRNVKER
Mexican cavefish              PVIADGAGGGDASSQAQPTGAVKGAPTEVVQPSDGRASLLESIRNAGGIGKAKLRNVKER
Electric eel                  PEFTDNTGAGDAASKAHPTGAVKGAPAEVLQPSDGRASLLESIRNAGGIGKAKLRNVKER
African clawed frog           LSIPAPAKKGGSDPGDQG--AVQGAPKEVVNPSNGRASLLESIRQAGGIGKANLRNVKEK
Western clawed frog           LPIPAPARVGSSDVGDPG--SLQGAPKEVVNPSDGRASLLESIRQAGGIGKAKLRNVKEK
Microcaecilia unicolor        LTSTPSQLGIDGGSDAAP---VQGAPKEVVNPSNGRASLLESIRQAGGIGKAKLRSVKEK
Chinese alligator             ALSKSPKAANEESSSAVPAAAVQGAPKEVVNPSTGRATLLESIRQAGGIGKANLRSVKER
Saltwater crocodile           ALSKSPKAASEESSSTVPAAAVQGAPKEVVNPSTGRATLLESIRQAGGIGKANLRSVKER
Chicken                       APSEPARTASEDSSKTVPAASVQGAPKEVVNPSTGRASLLESIRQAGGIGKANLRSVKER
Kakapo                        APSEPARTASEEESSSAVRAASVQGAPKEVVNPSTGRASLLESIRQAGGIGKANLRSVKER
Degu                          APTTGPGPLEDSSHGAQASAPVQGAPKEVVDPSSGRATLLESIRQAGGIGKAKLRSVKER
Tasmanian devil               IPAPAASEDSSSTCPSVP-------PKEVVDPSGGRATLLESIRQAGGIGKAKLRSVKER
Koala                         LTSAQPASEDNSS--MSPSVAVHVPPKEVVDPSSGRATLLESIRQAGGIGKAKLRSVKER
Golden hamster                TAPGPVAREEDAGGSVGHSASVQGAPKEVVDPSSGRASLLESIRQAGGIGKAKLRSVKER
Rat                           TVTGKVAREEDSGSGEAHSASVQGAPKEVVDPSSGRATLLESIRQAGGIGKAKLRSVKER
Mouse                         TAAGQVAREEDSSSSMAHTASVQGAPKEVVDPSSGRATLLESIRQAGGIGKAKLRSVKER
Blue whale                    APLGQPAREDD-SGGASP--SVQGAPKEVVEPSSGRATLLESIRQAGGIGKAKLRSVKER
American beaver               ASPAQGAREEDGTSSAPPSAPVQGAPKEVVDPSSGRATLLESIRQAGGIGKAKLRSVKER
Pig                           APPGQTAREDDSGGG-SPSVPVQGAPKEVVDPSSGRATLLESIRQAGGIGKAKLRSVKER
Panther                       ASPGQGAREEDRSGGLSP-ATVQGAPKEVVDPSSGRATLLESIRQAGGIGKAKLRSVKER
Cheetah                       ASPGQGAREEDRSGGLSP-ATVQGAPKEVVDPSSGRATLLESIRQAGGIGKAKLRSVKER
Dog                           ASPGQGAR-EDSSGSVSPSATIQGAPKEVVDPSSGRATLLESIRQAGGIGKAKLRSVKER
Monk seal                     ASLGQGAREDDSSGGVSPLATVQGAPKEVVDPSSGRATLLESIRQAGGIGKAKLRSVKER
Polar bear                    ASPGQGAR-EDSSGGVSPLATVQGAPKEVVDPSSGRATLLESIRQAGGIGKAKLRSVKER
European ferret               ASPVQGAREEDSSGGMTPLAAVQGAPKEVVDPSSGRATLLESIRQAGGIGKAKLRSVKER
Ugandan colobus               APVGQGARQDNSSS-ASPSAPVQGAPKEVVDPSGGRATLLESIRQAGGIGKAKLRSVKER
Chimpanzee chr20              APVGQGARQDDSSSSASP--SVQGAPREVVDPSGGRATLLESIRQAGGIGKAKLRSVKER
WASH1-20p13                   APVGQGARQDDGSSSASP--SVQGAPREVVDPSGGRATLLESIRQAGGIGKAKLRSVKER
WASHC1                        APVGQGARQDDSSSSASP--SVQGAPREVVDPSGGWATLLESIRQAGGIGKAKLRSMKER
WASHP4                        APVGQGARQDDSSSSASP--SVQGAPREVVDPSGGWATLLESIRQAGGIGKAKLRSMKER
WASHP6                        APVGQGARQDDGSSSASP--SVQGAPREVVDPSGGWATLLESIRQAGGIGKAKLRSMKER
                                                       * **::** *.*:******:*******:**.:**.

Zebrafish                     KMEKKKQKEQEQVGATVSGGDLMSDLFNKLAMRRKGISGKGPAGQGDSSEAPAS-----S
Mexican cavefish              KMEKKKQKEQEQVAVTASGGDFMSDLFNKLAMRRKGISGKVPGG-GDATEAPPS-----S
Electric eel                  KMEKKKQKEQEQVGATRSGGDFMSDLFNKLALRRKGISGKGPAG-SDATEAPAS-----S
African clawed frog           KLEKKKMKEQEQVGATGGGGDLMSDLFNKLAMRRKGISGKVPAA-GEAS----GDG---P
Western clawed frog           KLEKKKMKEQEQVRATGGGGDLMSDLFNKLAMRRKGISGKGPAA-GEAS----GDG---P
Microcaecilia unicolor        KMEKKKLKEQEQVRATG-GGDLMSDLFNKLAMRRKGISGKGPAV-GDGS----GDAPSTP
Chinese alligator             KLEKKKQKEQEQVRATGQGGDLMSDLFNKLVLRRKGISGKGPAAAGNAD-APGG-----P
Saltwater crocodile           KLEKKKQKEQEQVRATGQGGDLMSDLFNKLVLRRKGISGKGPAAAGNAD-APGG-----P
Chicken                       KLEKKKQKEQEQVRATGQGGDLMSDLFNKLVLRRKGISGKGPGA-SANPDAPGS-----P
Kakapo                        KLEKKKQKEQEQVRATGQGGDLMSDLFNKLVLRRKGISGKGPGASGNAD-APGS-----P
Degu                          KLEKKKQKEQEQVGAVGQGGDLMADLFNKLVLRRKGISGKGPGT-GANE----G-----P
Tasmanian devil               KLEKKKQKEQEQVRATSQGGDLMSDLFNKLVMRRKGISGKGPGA-STGE----G-----P
Koala                         KLEKKKQKEQEQVRATSQGGDLMSDLFNKLVMRRKGISGKGPGA-STGE----G-----P
Golden hamster                RLEKKKQKEQEQVRATSQGGDLMSDLFNKLVMRRKGISGKGPAS-GTSE----G-----P
Rat                           KLEKKKQKEQEQVRATSQGGDLMSDLFNKLVMRRKGISGKGPGT-GTSE----G-----P
Mouse                         KLEKKKQKEQEQVRATSQGGDLMSDLFNKLVMRRKGISGKGPST-GTSE----G-----P
Blue whale                    KLEKKKQKQQEQVRATSQGGDLMSDLFNKLAMRRKGISGKGPGP-GASE----G-----P
American beaver               KLEKKKQKEQEQVRATSQGGDLMSDLFNKLVMRRKGISGKGPGT-GTSE----G-----P
Pig                           KLEKKKQKEQEQARATSQGGDLMSDLFSKLAMRRKGISGKGPGP-GASE----G-----P
Panther                       KLEKKKQKEQEQVRATSQGGDLMSDLFNKLVMRRKGISGKGPAT-GTGE----G-----P
Cheetah                       KLEKKKQKEQEQVRATSQGGDLMSDLFNKLVMRRKGISGKGPAT-GTGE----G-----P
Dog                           KLEKKKQKEQEQVRATSQGGDLMSDLFNKLVMRRKGISGKGPVT-GASE----G-----P
Monk seal                     KLEKKKQKEQEQVRATSQGGDLMSDLFNKLVMRRKGISGKGPAT-GASE----G-----P
Polar bear                    KLEKKKQKEQEQVRATSQGGDLMSDLFNKLVMRRKGISGKGPAT-GASE----G-----P
European ferret               KLEKKKQKEQEQVRATSQGGDLMSDLFNKLVMRRKGISGKGPAT-GASE----G-----P
Ugandan colobus               KLEKKKQKEQEQVRATSQGGDLMSDLFNKLVMRRKGISGKGP---GAGE----G-----P
Chimpanzee Chr20              KLEKKKQKEQEQVRATSQGGDLMSDLFNKLVMRRKGISGKGP---GAGE----G-----P
WASH1-20p13                   KLEKKKQKEQEQVRATSQGGDLMSDLFNKLVMRRKGISGKGP---GAGE----G-----P
WASHC1                        KLEKQQQKEQEQVRATSQGGHLMSDLFNKLVMRRKGISGKGP---GAGE----G-----P
WASHP4                        KLEKQQQKEQEQVRATSQGGHLMSDLFNKLVMRRKGISGKGP---GAGD----G-----P
WASHP6                        KLEKKKQKEQEQVRATSQGGHLMSDLFNKLVMRRKGISGKGP---GAGE----G-----P
                              .:**:: *:***. ..  ** :*:***.**.:******** *   .       .     .
```

```
Zebrafish                   GGAFARMSDVIPPLPAPQQSAA-DDEDDWEA
Mexican cavefish            GSAFARMSDVIPPLPSPHQPTT-EDDDDWEA
Electric eel                GGAFARMSDVIPPLPAPQQPTT-EDDDDWEA
African clawed frog         TGAFARISDTIPPLPPPHQASGDGDEDDWES
Western clawed frog         TGAFARISDTIPPLPPPDQASGDGDEEDWES
Microcaecilia unicolor      GGAFARMSDTIPPLPPPQQPAGEENEDDWES
Chinese alligator           AGAFARVSDTIPPLPPPQQPPG-EDEDDWES
Saltwater crocodile         ANAFARVSDTIPPLPPPQQPPG-EDEDDWES
Chicken                     AGAFARMSDSIPPLPPPQQPPGEEDEDDWES
Kakapo                      AGAFARMSDSIPPLPPPQQPAGEEDEDDWES
Degu                        GGAFARVSDAIPPLPPPQQPAGEEDEEDWAS
Tasmanian devil             GGAFARMSDSIPPLPMLQQPPGEEDEDEWDS
Koala                       GGAFARMSDSIPPLPMLQQPPGEEDEDEWES
Golden hamster              GGAFTRMSDSIPPLPPPQQPAGDEDDDDWES
Rat                         GGAFSRMSDSIPPLPPPQQPAGDEDEDDWES
Mouse                       GGAFSRMSDSIPPLPPPQQPAGDEDEEDWES
Blue whale                  GGAFARMSDSIPPLPPLQPAPGEEDEDDWES
American beaver             GGAFARMSDSIPPLPPPQQPPGEEDEDDWES
Pig                         GGAFARMSDSIPPLPPP-QPPGEEDEDDWES
Panther                     GGAFARMSDSIPPLPPPQQPPGEEDEDDWES
Cheetah                     GGAFARMSDSIPPLPPPQQPPGEEDEDDWES
Dog                         GGAFARMSDSIPPLPPPQQPPGEEDEDDWES
Monk seal                   GGAFARMSDSIPPLPPPQQPPGEEEEDDWES
Polar bear                  GGAFARMSDSIPPLPPPQQPPGEEDEDDWES
European ferret             GGAFARMSDSIPPLPPPQQPPGEEDEDDWES
Ugandan colobus             GGAFARVSDSIPPLPPPQQPQAEEDEDDWES
Chimpanzee Chr20            GGAFARVSDSIPPLPPPQQPQAEEDEDDWES
WASH1-20p13                 GGAFARVSDSIPPLPPPQQPQAEEDEDDWES
WASHC1                      GGAFVRVSDSIPPLPPPQQPQAEEDEDDWES
WASHP4                      GGAFARVSDSIPPLPPPQQPQA-EDEDDWES
WASHP6                      GGAFARVSDSIPPVPPPQQPQAEEDEDDWES
                             .** *:** ***:*    .    ::::* :
```

Supplementary figure 1

Alignments of predicted *WASHC1* sequences from 27 vertebrate species with the 5 WASH1 genes annotated in UniProtKB (before the disabling mutations in the case of *WASH2P* and *WASH3P*) and with the new WASH1 gene annotated in the CHM13 assembly (WASH1-20p13). Where the human WASH1 proteins differ in sequence is highlighted in red. Disabling mutations (stop codons, frameshifts) are highlighted in yellow.
